# Supplementary material for: Machine Learning Accelerates Crystallization for Structure Determination
Source: Angew Chem Int Ed Engl. 2026 May 4;65(25):e1218503. doi: 10.1002/anie.1218503 (PMC13266958; doi:10.1002/anie.1218503)

checkCIF/PLATON report

Structure factors have been supplied for datablock(s) lcz049

THIS REPORT IS FOR GUIDANCE ONLY. IF USED AS PART OF A REVIEW PROCEDURE FOR PUBLICATION, IT SHOULD NOT REPLACE THE EXPERTISE OF AN EXPERIENCED CRYSTALLOGRAPHIC REFEREE.

No syntax errors found. CIF dictionary Interpreting this report

Datablock: lcz049

Bond precision:	C-C = 0.0087 A		Wavelength=1.54184
Cell:	a=8.5698(1)	b=24.9122(3)	c=14.9925(2)
	alpha=90	beta=101.814(1)	gamma=90
Temperature:	100 K		

	Calculated	Reported
Volume	3132.99(7)	3132.99(7)
Space group	C 2/c	C 1 2/c 1
Hall group	-C 2yc	-C 2yc
Moiety formula	C15 H3 Ag3 F18 N6, C10 H6 O2	C7.5 H1.5 Ag1.5 F9 N3, C5 H3 O
Sum formula	C25 H9 Ag3 F18 N6 O2	C12.50 H4.50 Ag1.50 F9 N3 O
Mr	1090.99	545.50
Dx,g cm-3	2.313	2.313
Z	4	8
Mu (mm-1)	16.274	16.274
F000	2080.0	2080.0
F000'	2090.25	
h,k,lmax	10,31,19	10,30,19
Nref	3365	3240
Tmin,Tmax	0.141,0.121	0.244,1.000
Tmin'	0.039	

Correction method= # Reported T Limits: Tmin=0.244 Tmax=1.000
AbsCorr = MULTI-SCAN

Data completeness= 0.963 Theta(max)= 78.300

R(reflections)= 0.0461(2984) wR2(reflections)= 0.1258(3240)

S = 1.107 Npar= 245

The following ALERTS were generated. Each ALERT has the format
test-name_ALERT_alert-type_alert-level.
Click on the hyperlinks for more details of the test.

Alert level C

```
PLAT041_ALERT_1_C Calc. and Reported SumFormula    Strings  Differ     Please Check
              Calc: C25 H9 Ag3 F18 N6 O2
              Rep.: C12.50 H4.50 Ag1.50 F9 N3 O
PLAT042_ALERT_1_C Calc. and Reported MoietyFormula Strings  Differ     Please Check
              Calc: C15 H3 Ag3 F18 N6, C10 H6 O2
              Rep.: C7.5 H1.5 Ag1.5 F9 N3, C5 H3 O
PLAT213_ALERT_2_C Atom F00G            has ADP max/min Ratio .....        3.2 prolat
PLAT213_ALERT_2_C Atom F00S            has ADP max/min Ratio .....        4.0 prolat
PLAT234_ALERT_4_C Large Hirshfeld Difference F00G     --C00Q     .       0.16 Ang.
PLAT342_ALERT_3_C Low Bond Precision on  C-C Bonds ..............    0.00869 Ang.
PLAT906_ALERT_3_C Large K Value in the Analysis of Variance ......      2.465 Check
PLAT911_ALERT_3_C Missing FCF Refl Between Thmin & STh/L=    0.600          6 Report
                1  1  0,  -6  0  2,  -7 21  4,  -7 21  5,   5 19  8,   2 12 15,
PLAT934_ALERT_3_C Number of (Iobs-Icalc)/Sigma(W) > 10 Outliers ..          1 Check
               -2 26  5,
```

Alert level G

```
PLAT003_ALERT_2_G Number of Uiso or U(i,j)  Restrained non-H-Atoms          3 Report
PLAT045_ALERT_1_G Calculated and Reported Z Differ by a Factor ...      0.500 Check
PLAT083_ALERT_2_G SHELXL Second Parameter in WGHT  Unusually Large      28.77 Why ?
PLAT186_ALERT_4_G The CIF-Embedded .res File Contains ISOR Records          1 Report
PLAT242_ALERT_2_G Low    'MainMol' Ueq as Compared to Neighbors of       C00N Check
PLAT242_ALERT_2_G Low    'MainMol' Ueq as Compared to Neighbors of       C00O Check
PLAT242_ALERT_2_G Low    'MainMol' Ueq as Compared to Neighbors of       C00Q Check
PLAT434_ALERT_2_G Short Inter HL..HL Contact F00C     ..F00C     .       2.74 Ang.
                                                2-x,y,3/2-z  =      2_756 Check
PLAT720_ALERT_4_G Number of Unusual/Non-Standard Labels ..........         33 Note
              Ag01    Ag02    F003    N004    F005    F006    O007    N008
              N009    F00A    F00B    F00C    C00D    H00D    C00E    C00F
              F00G    C00H    C00I    H00I    C00J    C00K    C00L    H00L
              C00M    H00M    C00N    C00O    C00P    H00P    C00Q    F00R
              F00S
PLAT860_ALERT_3_G Number of Least-Squares Restraints ............         18 Note
PLAT883_ALERT_1_G Absent Datum for _atom_sites_solution_primary ..     Please Do !
PLAT912_ALERT_4_G Missing # of FCF Reflections Above STh/L=  0.600        113 Note
PLAT941_ALERT_3_G Average HKL Measurement Multiplicity ..........        3.1 Low
PLAT969_ALERT_5_G The 'Henn et al.' R-Factor-gap value ..........      4.108 Note
              Predicted wR2: Based on SigI**2  3.06 or SHELX Weight 11.37
PLAT978_ALERT_2_G Number C-C Bonds with Positive Residual Density.          0 Info
```

```
   0 ALERT level A = Most likely a serious problem - resolve or explain
   0 ALERT level B = A potentially serious problem, consider carefully
   9 ALERT level C = Check. Ensure it is not caused by an omission or oversight
  15 ALERT level G = General information/check it is not something unexpected

   4 ALERT type 1 CIF construction/syntax error, inconsistent or missing data
   9 ALERT type 2 Indicator that the structure model may be wrong or deficient
   6 ALERT type 3 Indicator that the structure quality may be low
   4 ALERT type 4 Improvement, methodology, query or suggestion
   1 ALERT type 5 Informative message, check
```

Validation response form

Please find below a validation response form (VRF) that can be filled in and pasted into your CIF.

```
# start Validation Reply Form
_vrf_PLAT041_lcz049
;
PROBLEM: Calc. and Reported SumFormula    Strings  Differ     Please Check
RESPONSE: ...
;
_vrf_PLAT042_lcz049
;
PROBLEM: Calc. and Reported MoietyFormula Strings  Differ     Please Check
RESPONSE: ...
;
_vrf_PLAT213_lcz049
;
PROBLEM: Atom F00G            has ADP max/min Ratio .....        3.2 prolat
RESPONSE: ...
;
_vrf_PLAT234_lcz049
;
PROBLEM: Large Hirshfeld Difference F00G     --C00Q     .       0.16 Ang.
RESPONSE: ...
;
_vrf_PLAT342_lcz049
;
PROBLEM: Low Bond Precision on  C-C Bonds ..............    0.00869 Ang.
RESPONSE: ...
;
_vrf_PLAT906_lcz049
;
PROBLEM: Large K Value in the Analysis of Variance ......      2.465 Check
RESPONSE: ...
;
_vrf_PLAT911_lcz049
;
PROBLEM: Missing FCF Refl Between Thmin & STh/L=    0.600          6 Report
RESPONSE: ...
;
_vrf_PLAT934_lcz049
;
```

```
PROBLEM: Number of (Iobs-Icalc)/Sigma(W) > 10 Outliers ..          1 Check
RESPONSE: ...
;
# end Validation Reply Form
```

It is advisable to attempt to resolve as many as possible of the alerts in all categories. Often the minor alerts point to easily fixed oversights, errors and omissions in your CIF or refinement strategy, so attention to these fine details can be worthwhile. It is up to the individual to critically assess their own results and, if necessary, seek expert advice.

PLATON version of 17/09/2025; check.def file version of 17/09/2025

---

duplicate check

No duplication found

Datablock lcz049 - ellipsoid plot

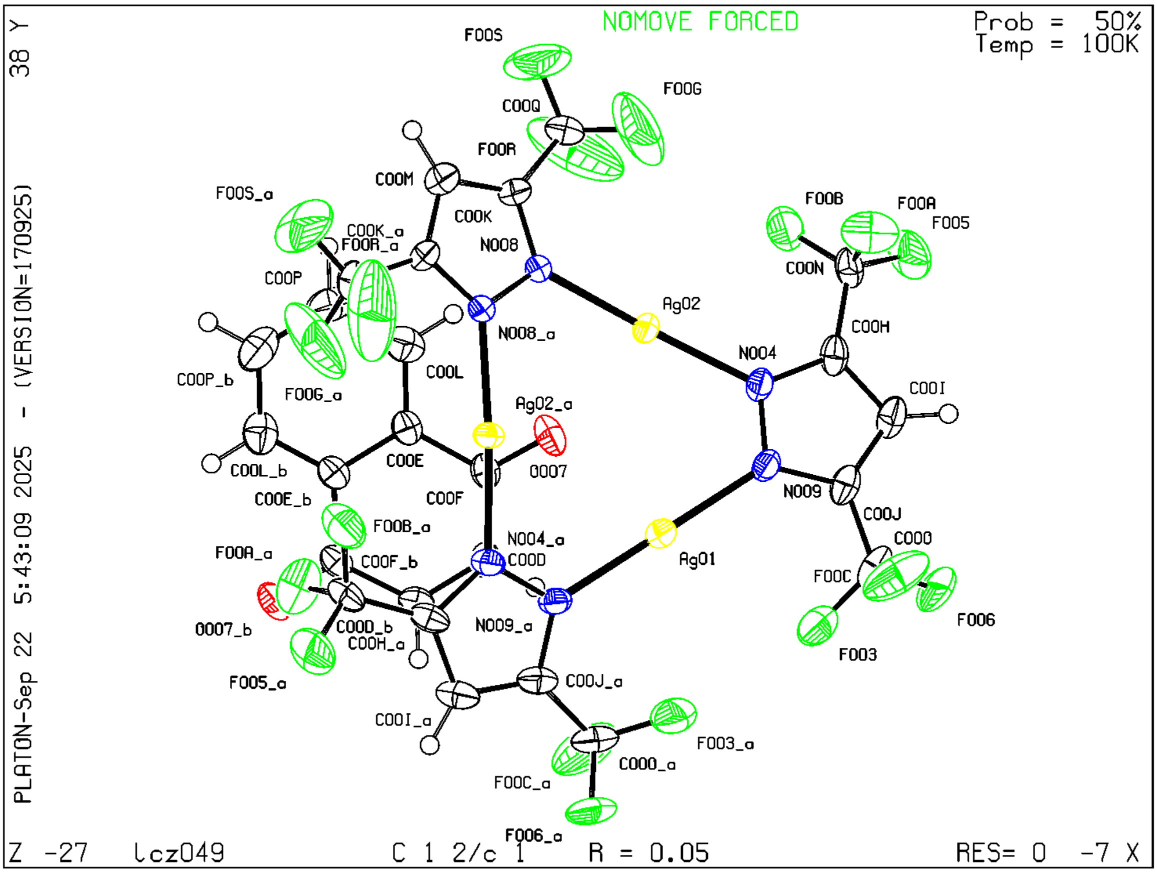

Supplement: Supplementary file 2 — Supporting File 2: anie72462‐sup‐0002‐DataFile.zip. [file ANIE-65-e1218503-s002.zip › supplementary_materials_crystallographic_data submit.zip!/cif_and_checkcif(1-114)/Ag3Pz3_49/Ag3Pz3_49_checkcif.pdf]
